# Ensemble feature selection and tabular data augmentation with generative adversarial networks to enhance cutaneous melanoma identification and interpretability

**DOI:** 10.1186/s13040-024-00397-7

**Published:** 2024-10-30

**Authors:** Vanesa Gómez-Martínez, David Chushig-Muzo, Marit B. Veierød, Conceição Granja, Cristina Soguero-Ruiz

**Affiliations:** 1 Department of Signal Theory and Communications, Telematics and Computing Systems, Rey Juan Carlos University, Madrid, 28943 Spain; 2 Oslo Centre for Biostatistics and Epidemiology, Department of Biostatistics, Institute of Basic Medical Sciences, University of Oslo, Oslo, Norway; 3 Norwegian Centre for E-health Research, University Hospital of North Norway, Tromsø, 9019 Norway

**Keywords:** Melanoma classification, Skin lesion classification, Ensemble feature selection, Tabular generative adversarial networks, Class imbalance, Interpretability methods

## Abstract

**Background:**

Cutaneous melanoma is the most aggressive form of skin cancer, responsible for most skin cancer-related deaths. Recent advances in artificial intelligence, jointly with the availability of public dermoscopy image datasets, have allowed to assist dermatologists in melanoma identification. While image feature extraction holds potential for melanoma detection, it often leads to high-dimensional data. Furthermore, most image datasets present the class imbalance problem, where a few classes have numerous samples, whereas others are under-represented.

**Methods:**

In this paper, we propose to combine ensemble feature selection (FS) methods and data augmentation with the conditional tabular generative adversarial networks (CTGAN) to enhance melanoma identification in imbalanced datasets. We employed dermoscopy images from two public datasets, PH2 and Derm7pt, which contain melanoma and not-melanoma lesions. To capture intrinsic information from skin lesions, we conduct two feature extraction (FE) approaches, including handcrafted and embedding features. For the former, color, geometric and first-, second-, and higher-order texture features were extracted, whereas for the latter, embeddings were obtained using ResNet-based models. To alleviate the high-dimensionality in the FE, ensemble FS with filter methods were used and evaluated. For data augmentation, we conducted a progressive analysis of the imbalance ratio (IR), related to the amount of synthetic samples created, and evaluated the impact on the predictive results. To gain interpretability on predictive models, we used SHAP, bootstrap resampling statistical tests and UMAP visualizations.

**Results:**

The combination of ensemble FS, CTGAN, and linear models achieved the best predictive results, achieving AUCROC values of 87% (with support vector machine and IR=0.9) and 76% (with LASSO and IR=1.0) for the PH2 and Derm7pt, respectively. We also identified that melanoma lesions were mainly characterized by features related to color, while not-melanoma lesions were characterized by texture features.

**Conclusions:**

Our results demonstrate the effectiveness of ensemble FS and synthetic data in the development of models that accurately identify melanoma. This research advances skin lesion analysis, contributing to both melanoma detection and the interpretation of main features for its identification.

**Supplementary Information:**

The online version contains supplementary material available at 10.1186/s13040-024-00397-7.

## Introduction

Cutaneous melanoma is the most aggressive form of skin cancer an important public health concern in fair-skinned populations. Globally, melanoma is projected to increase to 510000 new cases and 96000 deaths by 2040 [1]. Tumor thickness at diagnosis is the most relevant prognostic factor for localized melanoma, and early detection is crucial to effective clinical interventions and increased survival rates [2].

Dermoscopy is a noninvasive imaging technique that has substantially contributed to examining and identifying several skin lesions, including melanoma [3]. It involves the use of a dermatoscope, a device that uses optical magnification and cross-polarized lighting to capture magnified and illuminated skin images [4]. Dermoscopy images help to the visualization of pigmented structures within the epidermis and superficial dermis, supporting dermatologists to identify malignant skin lesions [4, 5]. These images have gained wide popularity in dermatological research owing to the availability of large public image datasets encompassing different types of skin lesions [6, 7]. Several studies have used images from these datasets to develop data-driven models capable of identifying skin lesions with high predictive performance [8, 9].

The advances of machine learning (ML) and deep learning (DL) have led to the development of models with high predictive performance in many domains [10]. Among these models, convolutional neural network (CNN)-based models have proven great performance in computer vision tasks, highlighting image classification [11]. CNN-based models and dermoscopy images have been employed in numerous studies in the literature to detect skin lesions [12]. Although CNNs have shown high predictive results, several authors have carried out a feature extraction process to capture underlying characteristics of skin lesions from dermoscopy images [13, 14]. While CNNs excel at automatically learning from images, feature extraction combined with traditional ML-based models offers several advantages [15]. Features extracted from dermoscopy images, such as color histograms, texture descriptors, or shape characteristics, can support the interpretability and clinical knowledge, helping to understand the key features involved in the model’s predictions. Among the main types of extracted features, geometric, color, and texture features have been extensively used [16]. Geometric features enable the identification of asymmetry, border irregularity, and diameter, which aids in distinguishing potentially malignant melanomas from benign lesions. Statistics from different color spaces has been shown to be useful for identifying skin lesions [15, 17]. Texture features allow us to capture the distribution and relationship between the gray pixel levels of skin lesions [16, 18–20]. While image feature extraction shows promise for detecting melanoma, extracting features from dermoscopy images of skin lesions often results in high-dimensional data that contains many irrelevant or redundant features [21, 22]. The high-dimensionality increases the computational cost for training models, augment the data sparsity, impact on the interpretability and hamper the performance of predictive models [21].

To address the main challenges associated with high-dimensionality, various methods have been proposed in the literature, with a particular emphasis on feature selection (FS) methods [23]. FS methods aim to select a subset of the most relevant features, thereby reducing the feature space and computational cost of training models, while also enhancing predictive performance and interpretability in subsequent tasks [24]. FS methods are categorized into three types, including filter, wrapper, and embedding methods [24], being filter methods the most extensively used due to the computational efficiency, ease of implementation and better generalization than wrapper and embedded methods [25]. Although the use of FS methods is extensive, a single FS method may generate local optimal or sub-optimal feature subsets, compromising the performance of subsequent predictive models [26]. To enhance the performance of traditional FS methods, recent studies have proposed the use of ensemble FS methods to select relevant features and improve the results in subsequent predictive tasks [27, 28]. The core idea behind ensemble FS is to combine multiple FS methods to identify the most relevant features. This approach offers several advantages over using a single FS method such as [29]: *i)* selecting feature sets that are more robust and less sensitive to data variations or limitations of any one FS method used, and *ii)* minimizing the risk of biases or limitations from any single FS method. Ensemble FS methods have been particularly effective in clinical studies [30, 31], where the identification of relevant features is critical. Thus, these methods may address the main challenges of high-dimensionality generated by feature extraction from dermoscopy images. This has a promising outlook, not only for selecting the most relevant geometric, color, and texture features for skin lesion detection but also for supporting interpretability.

In addition to the high-dimensionality, another of the main challenges in the development of ML/DL models is the presence of the class imbalance problem (CIP). The CIP occurs when the datasets have an unequal distribution of classes [6], *i.e.,* when the number of samples in one class (majority class) is significantly larger than other classes (minority classes) [32]. Regarding skin lesions, the majority of public image datasets (*e.g.,* ISIC, PH2, Derm7pt) presents a notable CIP, the scarcity of melanoma cases compared to benign skin lesions leads to severe CIP, making it difficult for ML-based models to learn distinguishing samples of the minority class (melanoma). Moreover, the variability in visual patterns and structures of melanoma (the atypical network, atypical streaks, atypical dots, and blue-white veil among others) can result in high intra-class variation, hampering the training of ML-based models. In classification tasks, the CIP can substantially affect the performance of ML models because it can lead to bias during learning algorithms, emphasizing the classification of samples belonging to classes with the greatest number of samples [33]. To overcome the CIP, data-level (resampling) methods have gained popularity, owing to their simplicity and ease of implementation. These methods balance a dataset by either generating new minority samples (oversampling approaches) or discarding the majority samples (undersampling approaches) [33]. However, these traditional approaches often have limitations, such as overfitting the minority class or losing valuable information by reducing the majority class.

Over the last years, oversampling approaches and more particularly the generative adversarial networks (GAN)-based models have gained considerable interest because of their capacity to create high-quality synthetic data, capturing the underlying structure of original data [34]. Several studies have used GAN-based models to augment the number of training samples and improve model performance [34, 35]. Despite their advantages, the majority of GANs are designed to work with images, and a few studies have explored the use of GANs to create synthetic samples in tabular data. Several methods have been proposed to create synthetic samples from tabular data, but most have pitfalls for mixed-type data (i.e.,, with both numerical and categorical features). Recently, the novel conditional tabular GAN (CTGAN) has shown excellent performance in addressing the main issues in the generation of mixed-type tabular data [36]. In the clinical setting, different authors have used CTGAN variants [37, 38] to create synthetic data that conserve underlying distribution from original data by aiming to enhance the results in predictive tasks. CTGAN is robust to handle datasets with both categorical and continuous variables since it uses mode-specific normalization to ensure that different types of data are accurately represented in the synthetic data [36]. Also, CTGAN has proven high effectiveness in multiple studies [39–42], and it has been benchmarked against other generative models [43–45], showing high performance in generating realistic synthetic tabular data.

The combination of ensemble FS methods and CTGAN is particularly advantageous for melanoma identification because it simultaneously tackles the issues of high-dimensionality and the CIP, which are relevant challenges for developing robust predictive models. In the literature, we identified studies that performed feature extraction for detecting skin lesions and employed different filter FS methods [19, 20, 46] and wrapper FS methods [13, 18, 47] to reduce dimensionality. However, these works only used a single FS method. Ensemble FS methods may offer significant advantages in melanoma detection, providing a more robust and stable selection of geometric, color, and texture features, and helping to build more generalizable and accurate predictive models. Regarding data augmentation, in dermatological research, we found previous studies that have tackled the CIP by using different image transformations (e.g., rotation, blurring, cropping) [13, 19, 47–49]. However, this data augmentation was carried out before the feature extraction, which can cause a notable bias in several shape, color, and texture characteristics of the skin lesions. Our approach, using CTGAN, that performs data augmentation using extracted features from skin lesions allows us to address these limitations and enhance the quality of synthetic data, thus favoring the clinical validity and increasing the performance of predictive models. By focusing on feature augmentation rather than image augmentation, we avoid the biases introduced by direct image transformations and ensure that the synthetic features accurately represent the true variability in the data [50]. This approach enhances the quality of synthetic data, thus favoring clinical validity and increasing the performance of predictive models.

In clinical research, interpretability in skin lesion classification is a critical aspect for the development of trustworthy and clinically applicable ML models [51]. Interpretability allows clinicians to gain insights into how and why a model makes certain predictions, fostering confidence in the model’s outputs, facilitating its integration into clinical practice, and leading to decision-making processes being transparent [52]. Although most of the current ML/DL models offer great predictive performance, these models lack interpretability, hampering their adoption and implementation in clinical practice. Explainable artificial intelligence (XAI) methods aim to provide transparency and interpretability to ML/DL models [53], being the post-hoc methods such as Shapley additive explanations (SHAP) [54] one of the most extended.

The aim of this study is two-fold. First, to evaluate the effectiveness of combining ensemble FS methods with GAN-based models to detect melanoma. Second, to identify the most relevant features for melanoma classification using interpretability methods, including SHAP, the bootstrap resampling statistical test [55] and visualizations with the uniform manifold approximation and projection (UMAP) [56]. To extract characteristics from skin lesions and obtain vector representations from dermoscopy images, two feature extraction approaches were used: *image features* and *image embeddings*. In the former, we extracted geometric features related to asymmetry, border and diameter [57], color features using different color spaces, and twelve techniques are used to extract texture features, mainly based on first-, second-, and high-order statistics. All of them will be detailed in next sections. In the latter case, ResNet-50 [58] was used to extract *image embeddings* from dermoscopy images. To address high-dimensionality, ensemble FS methods with Relief [59] as the *base FS learner* were considered to select relevant features and enhance melanoma classification using dermoscopy images. For data augmentation and to solve the CIP, our study focuses on quantifying the impact of aggregating new synthetic samples, controlled by the imbalance ratio (IR), on classification performance in imbalanced datasets. Data augmentation was performed using CTGAN on *image features* and *image embeddings* extracted from dermoscopy images belonging to two public skin datasets: Derm7pt [60] and PH2 [61]. To the best of our knowledge, this study is the first to explore ensemble FS methods combined with GAN-based models for tabular data augmentation using *image features* and *image embeddings* for melanoma classification. Additionally, through interpretability methods, our work contributes to the state-of-the-art by making an analysis of the most relevant geometric, color, and texture features involved in melanoma identification. This not only allows us to build accurate models, but it helps to understand which features are critical in identifying malignant lesions like melanoma, ensuring that predictive results are clinically meaningful.

## Related work

In this study, we conducted a feature extraction from dermoscopy images, encompassing geometric, color, and texture features, to identify skin lesions. The reviewed prior research has been mainly focused on works that have performed feature extraction and then we have selected those works that have addressed the high-dimensionality and the CIP using any FS method and data augmentation technique. In Table 1, we presented a summary of these studies, detailing the type of features extracted, the FS method and data augmentation technique used, the ML-based models employed, and the classification scenario.

**Table 1:** A summary of works that have used feature extraction and data augmentation for the detection of skin lesions. These works were listed from the most recent to appear to the oldest ones. N/A indicates ‘not applicable’

Ref.	Study	Extracted features	Feature selection	Data augmentation	Models used	Skin lesion
[48]	Sahoo et al. (2024)	Embeddings (ResNet50), LWT	NCA	Horizontal and Vertical flip, rotation	MLP, SVM, LR, DT, RF, KNN, NB	melanoma, not melanoma
[18]	Ghahfarrokhi et al. (2023)	Nonlinear indices (BCM, HFD, KFD, PFD) and texture (GLCM, DWT)	GA, PSO, WWO, NSGA-II	N/A	SVM, KNN, FitNet, FFNet, PatNet	melanoma, not melanoma
[49]	Shetty et al. (2022)	Color histogram, Haralick, Hu moments	N/A	Horizontal flip augmentation	CNN, DT, RF, SVM, KNN, LR, NB, LDA	seven skin lesions
[62]	Bansal et al. (2022)	Color, fractal signatures, texture (GLCM, LBP)	BHHO	Vertical/horizontal Flip, photometric and colorimetric changes	SVM	melanoma, not melanoma
[19]	Cheong et al. (2021)	Texture features based on entropy and energy	Student’s t-test	Rotation with 30, 60, 90	DT, LDA, QDA, SVM, KNN, PNN	melanoma, not melanoma
[63]	Ghalejoogh et al. (2020)	Shape, color, GLCM, lesion boundaries with CNDs	mRMR, SBFS	N/A	KNN, SVM, MLP, ENN ensemble classifier	melanoma, not melanoma
[47]	Chatterjee et al. (2019)	ABCD rule, fractal dimension, texture (GLCM, FRTA), color	RFE	Rotation, shifting	SVM	melanoma, not melanoma
[20]	Moura et al. (2019)	ABCD rule, GLCM, GLRLM, HOG, LBP, Tamura, box-counting VGG, CaffeNet	IGFS	N/A	MLP	melanoma, not melanoma
[16]	Khan et al. (2018)	Color, HOG features, texture (Haralick)	Entropy-variance method	N/A	DT, SVM, LR, NB, KNN, QDA, EBT	melanoma, not melanoma
[46]	Oliveira et al. (2017)	Shape, color, texture (DWT, (Fractal, GLCM)	IGFS, PCC, GRFS, PCA, Relief, CFS	RS	RF, AdaBoost, ensemble classifier	melanoma, not melanoma

Description of acronyms: lifting wavelet transform (LWT), naive Bayes (NB), multilayer perceptron (MLP), gray level co-occurrence matrix (GLCM), discrete wavelet transform (DWT), box-counting method (BCM), Higuchi fractal dimension (HFD), Katz fractal dimension (KFD), Petrosian fractal dimension (PFD), genetic algorithm (GA), particle swarm optimization (PSO), water waves optimization (WWO), fitting neural network (FitNet), feed-forward neural network (FFNet), pattern recognition network (PatNet), histogram of oriented gradients (HOG), local binary patterns (LBP), Harris hawks optimizer (BHHO), linear discriminant analysis (LDA), quadratic discriminant analysis (QDA), probabilistic neural network (PNN), complex network descriptors (CNDs), maximum relevance and minimum redundance (mRMR), sequential backward feature selection (SBFS), Elman neural network (ENN), fractal-based regional texture analysis (FRTA), recursive feature elimination (RFE), ensemble boosted tree (EBT), ensemble subspace discriminant analysis (ESDA), Pearson correlation coefficient (PCC), gain ratio-based feature selection (GRFS), principal component analysis (PCA), correlation-based feature selection (CFS), gray-level co-occurrence matrix (GLCM), gray-level run length matrix (GLRLM)

Most previous studies based on feature extraction for skin lesion classification have tackled the issue of high-dimensionality using two main types of FS approaches: *(i)* filter methods [19, 20, 46] and *(ii)* wrapper methods [13, 18, 47]. Among these works, some of them used both filter and wrapper methods individually [63]. Moreover, other studies used feature transformation methods such as NCA [48]. One of the primary gaps in prior research that we addressed is the lack of ensemble FS approaches to identify relevant features, including geometric, color, and texture features, for skin lesion classification. Existing works regarding cutaneous melanoma identification have explored the use of a single FS method [13, 16, 18–20, 46, 47, 63], which can be prone to bias and may not fully capture the optimal feature subset to identify skin lesions. Ensemble FS methods have gained significant attention for their ability to enhance the robustness of FS, and have proven to enhance the accuracy of subsequent predictive models in prior works [26, 64, 65]. It is worth noting that one of the contributions of this paper lies within the feature extraction, where we considered and fused an extensive variety of techniques to extract first-, second-, and high-order texture features, geometric and color features. The combination of these features not only seeks to improve the accuracy of lesion classification but also enhances the interpretability of skin lesion detection, providing an interpretable methodology for skin lesion classification, and bridging the gap between automated image analysis and clinical practice.

Regarding data augmentation, many methods have been proposed to cope with CIP, which can be categorized into data-level, algorithm-level and hybrid techniques [11]. Among these, data-level methods are extensively selected due to their ease of implementation and computational efficiency because they are independent of predictive models [66]. These methods reduce the skewed class distributions by either randomly discarding samples from the majority classes (undersampling approaches) or creating synthetic samples for minority classes (oversampling approaches) [67]. Although undersampling methods are easier to implement, these discard samples from the majority class, which can lead to a loss of information, and is particularly important when dealing with small datasets where every sample maintains valuable information. In this study, we mainly focus on oversampling approaches to address the CIP in skin lesion classification.

In computer vision applications, new versions of input images are created based on geometric transformations (horizontal/vertical flip), photometric changes (color jitter, gaussian blur), and colorimetric transformations (modifications of hue, saturation, and contrast) [68, 69]. In dermatological research, several studies for skin lesion classification have tackled the CIP in skin image datasets, primarily employing several image transformations such as rotation, blurring, and cropping to achieve class balance and increase the dataset size [13, 19, 47–49]. It is worth noting that several GAN-based models have been proposed to create synthetic images and combine them with original images with reasonable results [70–73], however these works used GANs for data augmentation and then CNN-based models for skin lesion identification.

## Dataset description and preprocessing

In this study, we employed dermoscopy images from two public datasets, PH2 [61] and Derm7pt [60]. These datasets have been extensively used in state-of-the-art research to evaluate the performance of models for skin lesion classification [74–76]. An overview of these datasets is shown in Table 2, which indicates the number and percentage of images per class and the corresponding IR.

**Table 2:** Overview of public dermoscopy image datasets used. The number and percentage of samples per class (‘melanoma’ and ‘not melanoma’ lesion), and the IR are shown

Dataset	# images	classes	% samples min./maj.	IR
PH2	200	‘not melanoma’ with 160 images, ‘melanoma’ with 40 images	20.0/80.0	4.00
Derm7pt	1,011	‘not melanoma’ with 759 images, ‘melanoma’ with 252 images	24.93/75.07	3.01

PH2 dataset consists of 200 dermoscopy images with 160 benign lesions (80 common nevi and 80 atypical nevi) and 40 melanoma [61]. The 160 common and atypical nevi were classified as ‘not melanoma’ and the 40 melanomas as ‘melanoma.’ Derm7pt dataset consists of 2,022 skin lesion images, with 1,011 dermoscopy images [60]. The dermoscopy images include 20 different skin lesion categories of which 14 are classified as ‘not melanoma’ (basal cell carcinoma, seborrheic keratosis, seven different nevi, and five miscellaneous lesions) and 6 as ‘melanoma’ (in situ, $<0.76$ mm, 0.76-1.5 mm, $>1.5$ mm, and metastatic melanoma) [60]. This resulted in 252 ‘melanoma’ and 759 ‘not melanoma’ images.

In these skin datasets, images may present two types of artifacts [15]: *acquisition artifacts*, such as air bubbles, ruler and ink marks, non-uniform illumination, and reflection[12], and *cutaneous artifacts*, including skin lines, blood vessels, and hair [12]. These artifacts not only hamper lesion segmentation and diagnosis but also hinder feature extraction. Among these artifacts, hair has a significant impact because its presence leads to occlusion of the texture, color, and boundary of the skin lesion [77]. To address this, a preprocessing stage was conducted, including image resizing, hair removal, and lesion segmentation. All dermoscopy images were resized to $224 \times 224$ pixels to reduce computational complexity and meet the input requirements of the pre-trained CNN-based models used (explained in the following sections). In the state-of-the-art, thresholding, clustering, and region-based techniques, and artificial neural networks (ANNs) have been explored for removing hair in dermoscopy images [78]. Owing to the remarkable results shown by ANNs and after a further review of the state-of-the-art, the novel model DoubleU-Net [79] was selected for skin lesion segmentation. An accurate segmentation is a crucial step previous to feature extraction because it allows to distinguish between the skin lesion and the surrounding healthy skin. Once a skin lesion is segmented, geometric, color, texture features can be extracted more effectively, focusing on characteristics from the lesion and discarding irrelevant information from surrounding skin.

DoubleU-Net, which combines two stacked U-Net architectures, has shown excellent results for performing lesion segmentation whereas eliminating image artifacts [79]. To assess the performance of DoubleU-Net on dermoscopy images, we conducted a quantitative evaluation using images from the ISIC-2016 dataset [80]. This dataset, with 900 dermoscopy images, includes binary masks that are useful for evaluating segmentation methods. The following segmentation metrics were used: Dice loss, Dice index, and intersection over union (IoU). By using five different partitions of ISIC-2016, DoubleU-Net achieved a Dice loss of 0.026±0.005, Dice index of 0.974±0.005, and IoU of 0.950±0.010.

## Proposed methodology

**Fig. 1:**
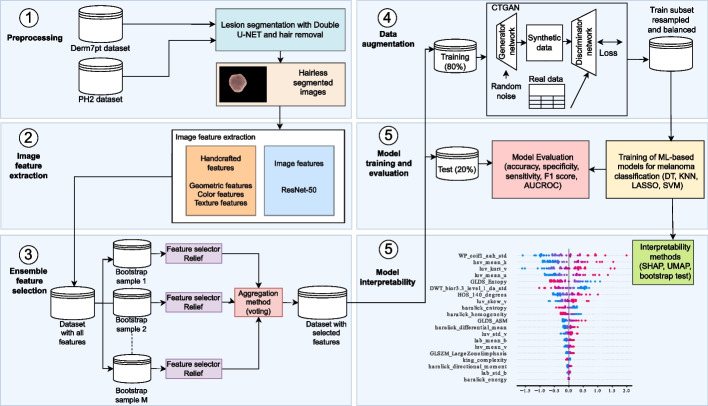
Workflow of the proposed methodology for melanoma identification

In this study, we developed a fully automated approach for detecting and classifying ‘melanoma’ and ‘not melanoma’ lesions using CNN-based and different ML-based models. Our approach consists of six stages: *(1) preprocessing, (2) feature extraction, (3) ensemble FS, (4) data augmentation, (5) model training, and evaluation, (6) model interpretability*. The stages involved in the proposed work are depicted in Fig. 1 and details about each stage are presented below.

1. *Preprocessing:* using dermoscopy images from PH2 and Derm7pt datasets, lesion segmentation with DoubleU-Net as well as the hair removal are performed.
2. *Feature extraction:* an automatic feature extraction is performed, considering handcrafted features and image embedding features to identify skin lesions. For the handcrafted features, we considered several geometric, color and texture features, whereas ResNet-50 was used to extract *image embeddings* from dermoscopy images.
3. *Ensemble FS:* because the feature extraction led to obtaining vectors with high-dimensionality, a dimensionality reduction is carried out using ensemble FS methods, with Relief as base selector.
4. *Data augmentation:* data augmentation is performed to address CIP and balance the dataset. CTGAN is considered as the oversampling method, generating synthetic samples for the minority class (in our case, ‘melanoma’).
5. *Model training and evaluation:* once the dataset was balanced, several ML-based models were used to classify between ‘melanoma’ and ‘not melanoma’ lesions, in particular the least absolute shrinkage and selection operator (LASSO), K-nearest neighbour (KNN), support vector machine (SVM), and decision tree (DT).
6. *Model interpretability:* to gain interpretability and identify the most important geometric, color, and texture features for melanoma classification, we considered SHAP, bootstrap resampling test, and UMAP.

### Image feature extraction

In this study, different *image features* were extracted from dermoscopy images to describe skin lesions. In the literature, *image features* are mainly categorized into handcrafted features (e.g., texture, color), dictionary-based features (e.g., bag-of-features), embedded features that use CNN-based models to automatically learn dense vector representations from images, and clinical features (used by physicians) that capture relevant characteristics for melanoma diagnosis [81]. We performed an automatic extraction of handcrafted and embedded features to identify ‘melanoma’ and ‘not melanoma’ lesions. For both handcrafted and embedded features, we first performed lesion segmentation using DoubleU-Net [79] and hair removal using morphological operations and fast marching inpainting [82]. For handcrafted features, after preprocessing, we started with a comprehensive set of geometric, color, and texture features. Ensemble FS methods were then employed to reduce dimensionality and select the most relevant features. For embedded features, after applying the same preprocessing steps, we used ensemble FS methods to reduce dimensionality.

The CNN-based model ResNet-50 [58] was used to extract *image embeddings* from dermoscopy images, which have been extensively used in the literature because it uses residual blocks with shortcut connections to solve performance degradation and vanishing gradient [58, 83]. Although several ResNet variants with different numbers of layers (18, 34, 50, 101, and 152) have been proposed [58], ResNet-50 was considered because of its proven predictive performance and computational efficiency (as demonstrated in previous studies [9, 58]). Transfer learning and fine-tuning techniques were used to improve the extracted *image embedding*. First, a pretrained model $\mathcal {M}_{model}$ with layers $L=\{l_1, \dots , l_n\}$ is trained using a large dataset $\mathcal {D}_{large}$ (in our case, ImageNet [84]). Then, we trained a new model $\mathcal {M}^{\prime }_{model}$ using the following steps: (1) freezing the preceding layers *L* of $\mathcal {M}_{model}$; (2) adding new layers to *L* and creating a new architecture with $L^{\prime }=\{l_1, \dots , l_{n-1}\} \cup \{l^{\prime }_1, \dots , l^{\prime }_n\}$; and (3) training the added layers of $\mathcal {M}^{\prime }$ on a new dataset $\mathcal {D}^{\prime }_{dermo}$ (either the PH2 or Derm7pt dataset). A high-dimensional vector was extracted from the final global average pooling layer by retaining the convolutional and pooling layers responsible for image feature extraction. Each image yielded a vector comprising 2,048 features (*image embedding*).

For handcrafted features, we included geometric features, dermoscopic features, color, and local/global texture features. An overview of the different types of features extracted from dermoscopy images is presented in Table 3. Among the main approaches to aid dermatologists in the identification of melanoma, a commonly used geometric rule is the ABCD rule [57]. Automatic feature extraction was performed using the ABCD rule.

**Table 3:** Summary of image features extracted from segmented skin lesions

Feature	Technique	Studies	# features	Selected features
Geometric	ABCD	[85–87]	4	assymetry (geo_assymetry), border (geo_border), color (geo_color), diameter (geo_diameter)
Texture	DWT	[88, 89]	34	DWT features. Examples: DWT_bior3.3_level_da_mean
	FDTA	[90]	4	FDTA Hurst coefficients (FDTA_HurstCoeff_1, FDTA_HurstCoeff_2, FDTA_HurstCoeff_3, FDTA_HurstCoeff_4)
	FOS	[86, 91]	15	energy (fos_energy), mean (fos_mean), std (fos_std), median (fos_median), variance (fos_variance), mode (fos_mode), skewness (fos_skew), entropy (fos_entropy), maximal gray level (fos_mxgl), coefficient of variation (fos_cov), minimal gray level (fos_mngl) percentiles (fos_10, fos_25, fos_75, fos_90)
	GLCM	[13, 14, 47]	14	angular second moment (glcm_asm), contrast (glcm_contrast), sum of squares (glcm_sos), inverse difference moment (glcm_idm), sum average (glcm_sa), correlation (glcm_corr), sum variance (glcm_sv), sum entropy (glcm_se), entropy (glcm_entropy) difference variance (glcm_dv), difference entropy (glcm_de)
	GLDS		5	homogeneity (glds_H), contrast (glds_C), mean (glds_M), entropy (glds_EN), mode (glds_mode), skewnewss (glds_skew), minimal gray level (glds_mngl), maximal gray level (glds_mxgl), coefficient of variation (glds_cov), energy (glds_energy), entropy (glds_entropy), histogram width (glds_hw) percentiles (10, 25, 75, 90), kurtosis (glds_kurt)
	GLRLM	[92, 93]	16	short run emphasis (GLRLM_sre), long run emphasis (glrlm_lre), gray level non-uniformity (GLRLM_GrayLevelNo-Uniformity), run percentage (glrlm_rp), run length non-uniformity/run Length distribution (glrlm_rlnu), high gray level run Emphasis (glrlm_hglre), low gray level run emphasis (glrlm_lglre), short run high gray level emphasis (glrlm_srhgle), long run low gray level emphasis (glrlm_sre), long run high gray level emphasis (glrlm_sre), short low gray level emphasis (glrlm_sre)
	GLSZM	[94, 95]	14	small zone emphasis (glszm_sze), large zone emphasis (glszm_lze), gray level non-uniformity (glszm_glnu), zone-size non-uniformity (glszm_zsnu), low gray level zone emphasis (glszm_lglze), zone percentage (glszm_zp), high gray level zone emphasis (glszm_hglze), small zone low gray level emphasis (glszm_szlgle), small zone high gray level emphasis (glszm_szhgle), large zone low gray level emphasis (glszm_lzlgle), large zone high gray level emphasis (glszm_hglze), gray level variance (glszm_szhgle), zone-size variance (glszm_zsvar), zone-size entropy (glszm_zsentr)
	HOS	[96]	2	HOS at 135 degrees (HOS_135_degrees), HOS at 140 degrees (HOS_140_degrees)
	King	[97]	5	coarseness (king_coarseness), contrast(king_contrast), complexity(king_complexity), strength (king_strength), busyness(king_busyness)
	LBP	[98, 99]	6	energy (LBP_R_1_P_8_energy, LBP_R_2_P_16_energy, LBP_R_3_P_24_energy) entropy (LBP_R_1_P_8_entropy, LBP_R_2_P_16_entropy, LBP_R_3_P_24_entropy)
	SFM		4	coarseness (SFM_Coarseness), contrast (SFM_Contrast), periodicity (SFM_Periodicity), roughness (SFM_Roughness)
	WP	[100, 101]	125	WP features. Examples: WP_coif1_aah, WP_coif1_aav, WP_coif1_aad),
Color	RGB	[13, 62]	4	rgb_mean, rgb_std, rgb_skewness, rgb_kurtosis
	HSV	[13, 62, 102]	4	hsv_mean, hsv_std, hsv_skewness, hsv_kurtosis
	CIE L*a*b	[13, 62]	12	The mean, std, skewness, and kurtosis were computed for each channel.
	CIE L*u*v	[103, 104]	12	The mean, std, skewness, and kurtosis were computed for each channel.
	YCrCb	[13, 62, 103]	12	The mean, std, skewness, and kurtosis were computed for each channel.

Several studies have investigated the effectiveness of color and texture features for identifying skin lesions in dermoscopy images [15, 17, 105]. Regarding color, RGB (red, green, blue) is the most well-known color space, but it has several drawbacks. It is not perceptually uniform and presents a high correlation between channels [15]. This motivated the use of other color spaces to extract color information from skin lesions [17]. HSV is composed of hue (H), saturation (S), and value (V) components, which distinguish between luminance and chrominance. The YCbCr color space represents chromaticity components according to luminance (Y), blue difference (Cb), and red difference (Cr). Both CIE L*a*b and CIE L*u*v were proposed to provide a uniform color space [106], where the computation of luminance (L) and chroma (a*b or u*v) is obtained through a nonlinear mapping of the XYZ coordinates. To characterize the color distribution in skin lesions, we considered the RGB, HSV, CIE L*a *b, CIE L*u *v, and YCbCr color spaces. The mean, standard deviation (std), skewness, and kurtosis were computed for each channel of the color spaces.

Texture features provide quantitative information on the distribution of pixel intensity levels within a region of interest of an image [107, 108], thereby supporting skin lesion identification. The main methods for capturing textures are categorized into statistical, signal-processing, geometrical, and model-based approaches [109]. We focus on statistical methods that calculate the gray-level histogram of an image and capture information about the distribution and frequency of pixels with specific intensity within a region of interest. The following groups of features were extracted from the dermoscopy images: discrete wavelet transform (DWT), fractal dimension texture analysis (FDTA), first-order statistics (FOS), gray-level co-occurrence matrix (GLCM) or Haralick features, gray-level difference statistics (GLDS), gray-level run length matrix (GLRLM), gray-level size zone matrix (GLSZM), higher-order spectra (HOS), King features, local binary patterns (LBP), statistical feature matrix (SFM), and wavelet packet (WP) decomposition features.

DWT relies on wavelet band superposition, enabling multi-resolution analysis, and the values in the sub-band images (or combinations) capture texture information [110]. FDTA is based on the calculation of Hurst coefficients for capturing the roughness of an image [111]. FOS features describe the distribution of pixel-level intensities within a region of interest [112]. GLCM are second-order features that quantify the spatial relationships that occur in neighboring pixels with similar (or specific) intensity within an image [113]. GLDS are first-order features that measure the absolute differences in the gray level between two pixels separated by a displacement vector [114], thereby capturing the correlation degree of different pixels in the neighborhood and assessing the heterogeneity of a region of interest. GLRLM extracts higher-order texture features that measure the gray intensity pixels in a particular direction from a reference pixel [115, 116]. GLSZM features not only capture information of gray-level intensities but also measure the connectedness of the gray-level intensities [117].

HOS features extract complex patterns in the distribution of grayscale levels, with the third-order spectrum (bispectrum) being the most used [118]. LBP extracts the local texture features by analyzing the center pixel intensity with its neighboring pixels [13]. King features use a neighborhood gray-tone difference matrix to capture textures from images [119]. SFM measures the statistical properties of pixel pairs at several distances within an image [120]. WP represents a generalization of multiresolution analysis and uses sub-band decompositions to capture texture information [121].

### Ensemble feature selection methods

**Fig. 2:**
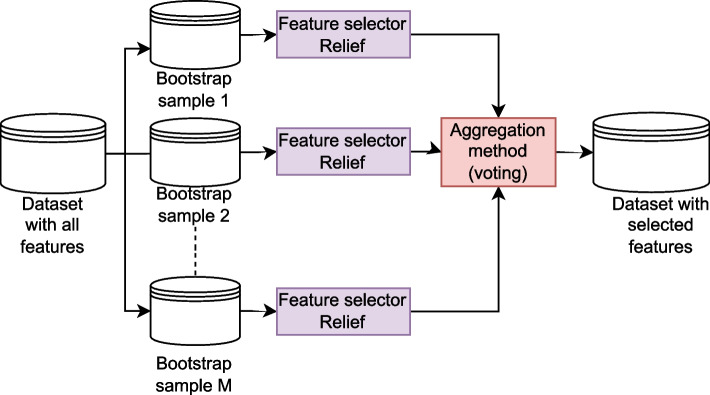
A schematic diagram of homogeneous ensemble FS

FS methods select the most relevant features with the aim of improving the performance of subsequent predictive models [24] and are mainly categorized into three categories: filter, wrapper, and embedding methods [24]. Ensemble FS has been recently studied to enhance the performance of traditional FS methods [27, 28]. Ensemble FS methods combine multiple *base FS learners* by selecting different sets of selected features to produce a robust selection of features. Given a training subset $\mathcal {X}_{train}=\{\textbf{x}^{(i)}\}_{i=1}^{n}$ consisting of *n* samples, the *i*th sample is represented by a vector $\textbf{x}^{(i)}=[x_{1}^{(i)}, \dots , x_{D}^{(i)}] \in \mathbb {R}^D$, where *D* is the number of features. *M* new training subsets are created (each of approximate size $n_{train}$) by sampling uniformly and with replacement $\mathcal {D}_{train}$. These new versions of the training subsets are known as *bootstrap subsets*, $\mathcal {X}_{boot}$. A total of $\{ \mathcal {X}_{boot}^{(j)} \}_{j=1}^{M}$ are generated, and *M* *base FS learners* are trained using these bootstrap subsets and assign an importance value to each feature. A feature importance vector $\textbf{v}^{(j)}=[v_{1}^{(j)}, \dots , v_{D}^{(j)}] \in \mathbb {R}^D$ is obtained for each $\mathcal {X}_{boot}^{(j)}$ with a total of $\{ \textbf{v}^{(j)} \}_{j=1}^{M}$ vectors. Finally, these feature importance vectors are combined to obtain a robust selection that leads to better predictive performance. A crucial stage in ensemble FS is the combination of features selected by *base FS learners*. In this study, rank aggregation based on voting was used [28, 122]. A schematic of the ensemble FS is shown in Fig. 2.

Recent studies have shown that ensemble FS methods improve the predictive performance [123]. Ensemble FS methods are classified based on the type of *base FS learners* into homogeneous and heterogeneous approaches [122]. In homogeneous approaches, the same *base FS method* is employed for different *bootstrap subsets*, whereas various FS methods are considered in heterogeneous approaches. In this study, we utilized a homogeneous ensemble approach with Relief as *base-FS learners*.

### Tabular data augmentation through generative adversarial networks

GANs, originally proposed by Goodfellow [124], are generative models based on a competitive learning and composed of two ANNs: a generator *G* and a discriminator *D*. *G* takes a random vector $\textbf{z}$ from a distribution $F_z \sim \mathcal {N}(0, 1)$ and projects it to a vector $\mathbf {\hat{x}}$, while the discriminator *D* seeks to distinguish between real data and synthetic data. During the competitive learning, the generator *G* seeks to generate indistinguishable synthetic data from real data, whereas discriminator *D* is trained to discriminate whether a sample is real or synthetic. *G* and *D* aim to optimize a zero-sum min-max game, with the value function *V*(*G*, *D*) as follows:

$$\begin{aligned} \min _{G} \max _{D} V(G, D)~=~\mathbb {E}_{x \sim \rho _{data}(x)}[logD(\textbf{x})]+\mathbb {E}_{z \sim \rho _{z}(z)}[log(1 - D(G(\textbf{z}))) \end{aligned}$$

where $\rho _{data}(x)$ and $\rho _{z}(z)$ are the distribution of the real data and that of the samples generated by *G*, respectively. $\textbf{x}$ and $\textbf{z}$ represent the samples from the input and the latent space, and $E_x$ and $E_z$ are the expected log-likelihood from the different outputs of both real and generated samples.

Despite the remarkable results of GANs, these models are typically optimized for creating synthetic images or texts. CTGAN was designed for generating tabular data, and particularly when datasets are composed of categorical and continuous features (mixed-type data) [36]. To effectively generate tabular data, CTGAN introduces several changes in its architecture, including conditional generation, mode-specific normalization and non-satured loss functions. The conditional generation involves conditioning the generation process on specific values of categorical variables. This ensures that the generator produces samples that are representative of specific subpopulations within the data. The mode-specific normalization aim to overcome the features with non-Gaussian and multimodal distributions. Each column is processed independently and each value is represented as a one-hot vector indicating the mode and a scalar the value within the mode. Three steps are followed to achieve this: *(i)* for each continuous column $C_i$, a variational Gaussian model (VGM) is used to estimate the number of modes $m_i$ and fit a Gaussian mixture; *(ii)* for each value $c_{i,j}$ in $C_i$, the probability of $c_{i,j}$ of belonging to each mode is computed; and *(iii)* a mode is chosen based on the given probability density, and then use the chosen mode to normalize the value. Then, $c_{i,j}$ is represented as a one-hot vector.

To evaluate the similarity between synthetic and real data and to quantitatively assess the quality of synthetic samples, several quality metrics have been proposed in the literature [38, 125]. To measure the univariate attribute fidelity, we used the Hellinger distance (HD) [126], whereas to assess how well CTGAN captures the relationships between features, the pairwise correlation difference (PCD) [125] was considered. We additionally used two metrics proposed by the authors in [38], the mean absolute error probability (MAEP) and repeated sample vector rate (RSVR).

Given a particular feature *x* present in both the real dataset $\mathcal {D}_{real}$ and the synthetic dataset $\mathcal {D}_{syn}$, we computed the corresponding probability mass functions for the real data, denoted as $P_x$, and the synthetic data, denoted as $Q_x$. Using these distributions, we define the following quality metrics:

The HD measures the similarity between $P_x$ and $Q_x$ as follows [126]:

$$\begin{aligned} HD(P_x, Q_x) = \frac{1}{\sqrt{2}} \sqrt{\sum _{j=1}^{k_x} \left( \sqrt{P_x(j)} - \sqrt{Q_x(j)} \right) ^2} \end{aligned}$$

where $k_x$ represents the number of categories for the feature *x*. HD is ranged from 0 to 1, with 0 indicating that the distributions are similar, and 1 maximum divergence.

PCD quantifies the difference between the correlation matrices associated with the real and synthetic data, and identified as $\mathcal {X}_{real}$ and $\mathcal {X}_{syn}$. PCD is calculated as follows:

$$\begin{aligned} PCD(X_{real}, X_{syn})=\Vert Corr(X_{real}) - Corr(X_{syn})\Vert \end{aligned}$$

where $Corr(\cdot )$ denotes the correlation matrix, and $\Vert \cdot \Vert$ represents the matrix norm that yields a scalar reflecting the degree of similarity between the two correlation matrices. Lower PCD values suggest that the relationships between features in $\mathcal {X}_{real}$ are better preserved in $\mathcal {X}_{syn}$ [125].

MAEP evaluates the absolute difference between two PMFs, and for a given feature x, the MAEP is defined as follows:

$$\begin{aligned} MAEP(P_x, Q_x)=\sum _{j=1}^{k_x} \vert P_x(j) - Q_x(j) \vert \end{aligned}$$

where $k_x$ represents the number of categories for the feature *x*.

RSVR quantifies the occurrence of duplicate sample vectors in the synthetic dataset $\mathcal {X}_{syn}$, indicating how well the oversampling technique generates unique vectors. Note that RSVR is influenced by the number of synthetic samples generated, as the IR increases, the likelihood of repeated vectors also rises, leading to higher RSVR values.

### Interpretability methods

To gain interpretability of the color, texture, and geometric features (*image features*) most relevant for identifying skin lesions, we employed three different methods: SHAP [54], confidence intervals with bootstrap resampling [127], and UMAP [56].

SHAP relies on game theory, combining optimal credit allocation and local explanation to compute the contribution of each feature to model predictions using Shapley values [54]. The Shapley value quantifies the mean marginal contribution of each feature across all possible subsets of features. In the context of binary classification, such as distinguishing between ‘melanoma’ and ‘not melanoma’ lesions, the Shapley value for a feature $x_i$ is defined as:

$$\begin{aligned} \phi _i(f, \textbf{x}) = \sum _{S \subseteq \{x_1, \dots , x_D\} \setminus \{x_i\}} \frac{|S|!(D - |S| - 1)!}{D!} \left[ f(S \cup \{x_i\}) - f(S) \right] \end{aligned}$$

where *D* is the total number of features, *S* is a subset of features that does not include $x_i$, and *f*(*S*) and $f(S \cup \{x_i\})$ are the model predictions without and with the feature $x_i$, respectively. This formula calculates the contribution of each feature to the final prediction of whether a lesion is ‘melanoma’. The absolute value of the Shapley value $|\phi _i(f, \textbf{x})|$ reflects the significance of the feature’s contribution, allowing us to rank features based on their impact on the model’s decision-making process. SHAP ranks features according to their contribution to the model’s predictions, providing a clear and interpretable explanation of how each feature influences the classification of lesions as ‘melanoma’ and ‘not melanoma’.

To identify the most relevant geometric, color, and texture features associated with ‘melanoma’ and ‘not melanoma’ lesions, we used the confidence intervals with bootstrap resampling [127], which allow to measure the distribution of a statistic (mean for numerical features, and proportion for binary features) on a population using resamples. In our case, we compute the difference between the features associated with the ‘melanoma’-population $\mathcal {X}_{melanoma}$ and the ‘not melanoma’-population $\mathcal {X}_{not melanoma}$. The bootstrap resampling was applied $M=1000$ times, resampling the original dataset and obtaining *M* subset versions for each class, i.e., $\{ \mathcal {X}_{melanoma}^{(i)} \}_{i=1}^{M}$ and $\{ \mathcal {X}_{not melanoma}^{(i)} \}_{i=1}^{M}$ for $\mathcal {X}_{melanoma}$ and $\mathcal {X}_{not melanoma}$, respectively. We assessed the difference $\Delta$ between $\mu _{melanoma}$ (the statistic of a feature in $\mathcal {X}_{melanoma}$) and $\mu _{not melanoma}$ (the statistic of the same feature in $\mathcal {X}_{not melanoma}$), i.e., $\Delta =\mu _{melanoma}-\mu _{not melanoma}$. Subsequently, we computed the *M* differences $\{ \mu _{melanoma}^{(i)} \}_{i=1}^{M}$ and $\{ \mu _{not melanoma}^{(i)} \}_{i=1}^{M}$, and the difference between the statistic in both populations $\Delta ^{(i)}=\mu _{melanoma}^{(i)}-\mu _{not melanoma}^{(i)}$. Lastly, we estimated the 95% confidence interval ($CI_\Delta$) for each feature and a statistical hypothesis test is conducted, where the null hypothesis $\mathcal {H}_{0}$ is true if $0 \in CI_\Delta$, whereas the alternative hypothesis $\mathcal {H}_{1}$ is considered true if $0 \not \in CI_\Delta$ (e.g.,, no overlapping with 0). When $\mathcal {H}_{1}$ is met, it indicates a statistically significant difference between the mean/proportion of a specific feature in ‘melanoma’ and ‘not melanoma’ lesions.

To visually compare the *image features* associated with ‘melanoma’ and ‘not melanoma’ lesions, the nonlinear dimensionality reduction technique called UMAP [56] was used. UMAP is a nonparametric dimensionality reduction technique that allows the visualization of high-dimensional data into a low-dimensional space while maintaining the structure of the original data [56]. UMAP technique is designed to maintain the local and global structure of data, and allow us to visualize data into a low-dimensional space to reveal hidden patterns [56].

## Experiments and results

### Experimental setup

In this study, different ML models were considered for the classification of melanoma lesions. We compared the performance of LASSO, KNN, SVM, and DT [128, 129]. To evaluate the predictive performance, we considered the sensitivity, specificity, and the area under the receiver operating characteristic curve (AUCROC) [129]. *k*-fold cross-validation [128] with $k=5$ was employed to determine the optimal hyperparameters for the ML models, using AUCROC as the figure of merit. AUCROC was selected because it effectively captures the performance of ML models on datasets with an uneven class distribution. The following hyperparameters were explored: *C* in the range $[1e^{-1.5}, 1e^{0.4}]$ for LASSO, *K* between [1, 11] for KNN, and $\gamma \in \{1e^{-2}, 1e^{-3}, 1e^{-4}, 1e^{-5}\}$ and *C* in the range $[1e^{-0.9}, 1e^{0.9}]$ for SVM. For DT, the maximum depth in the range [2, 8] and the minimum samples per split are dynamically determined based on the size of the training set. Oversampling methods were applied five times, each with varying IR values. For each iteration, the ML models were independently trained and tested on different train-test subsets to obtain generalizable predictive results. The performance was evaluated by computing the mean and standard deviation across these repeated experiments. The source code for the reproducibility of results is available in the Github repository: github.com/ai4healthurjc/ensemble-fs-aug-melanoma.git.

To extract embeddings from dermoscopy images, several ResNet-50 models were trained specifically for binary classification tasks. After the training process, embeddings were extracted from the final global average pooling layer of these models. The binary cross-entropy loss function, suitable for binary classification, was used as the cost function, and the Adam optimizer [130] was selected to ensure efficient convergence. The batch size was determined based on the dataset size, with specific values chosen for different datasets: a batch size of 32 for Derm7pt and 8 for the PH2 dataset. An adaptive learning rate strategy was implemented, gradually reducing the learning rate during training. Additionally, early stopping was applied after 15 epochs to prevent overfitting [130].

### Quality evaluation of synthetic data

**Fig. 3:**
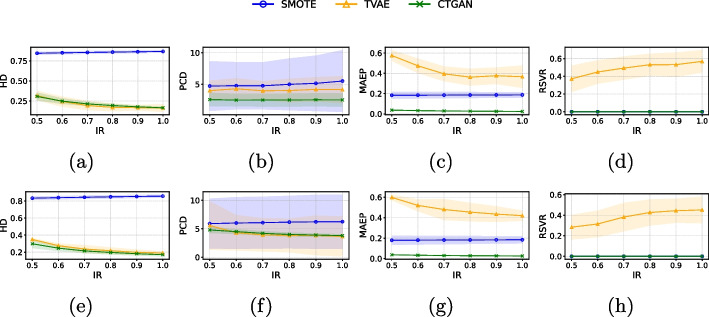
Mean ± standard deviation of the quality metrics (HD, PCD, MAEP, RSVR) of generated synthetic samples for *image features* (first row) and *image embeddings* (second row) in the PH2 dataset when considering SMOTE, TVAE and CTGAN

**Fig. 4:**
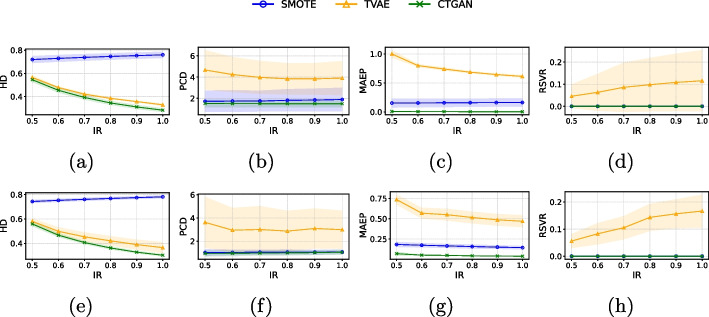
Mean ± standard deviation of the quality metrics (HD, PCD, MAEP, RSVR) of generated synthetic samples for *image features* (first row) and *image embeddings* (second row) in the Derm7pt dataset when considering SMOTE, TVAE and CTGAN

Several data quality metrics were considered to evaluate the similarity between synthetic and real data across different IR values (see Figs. 3 and 4). We compared CTGAN with established state-of-the-art methods, including the synthetic minority oversampling technique (SMOTE) [131] and the tabular variational autoencoder (TVAE) [132].

For the PH2 dataset, in both *image features* (see Fig. 3a, b) and *image embeddings* (see Fig. 3c, d), CTGAN demonstrated superior performance compared to the oversampling methods SMOTE and TVAE in terms of HD, MAEP, RSVR and PCD. These quality metrics highlight that CTGAN excels in preserving the correlation between features while generating probability distributions that are more similar between real and synthetic data. Moreover, in terms of HD, CTGAN outperformed SMOTE while maintaining comparable performance to TVAE (see Fig. 3a for *image features* and Fig. 3e for *image embeddings*). Regarding the RSVR (see Fig. 3d for *image features* and Fig. 3h for *image embeddings*), which identifies the percentage of repeated vectors generated by the oversampling methods, it was observed that CTGAN and SMOTE did not generate repeated vectors, whereas TVAE did. For Derm7pt dataset (see Fig. 4), in both *image features* and *image embeddings*, we observed that, in terms of HD, PCD and MAEP (see Fig. 4a-c for *image features*, and Fig. 4e-g for *image embeddings*), CTGAN demonstrated superior performance compared to SMOTE and TVAE. In terms of RSVR (Fig. 4d for *image features*, and Fig. 4h for *image embeddings*), similar to the results observed with the PH2 dataset, it was found the CTGAN and SMOTE did not generate repeated vectors. Thus, it is observed that CTGAN is a promising method for generating synthetic data, outperforming other oversampling methods in different quality metrics analyzed (PCD and MAEP) while maintaining parity in the remaining two (RSVR and HD) for the PH2 dataset. For the Derm7pt dataset, CTGAN is the best performer in three of the four quality metrics (HD, PCD and MAEP) and remains equal in RSVR.

### Melanoma classification by combining ensemble FS methods and data augmentation

**Fig. 5:**
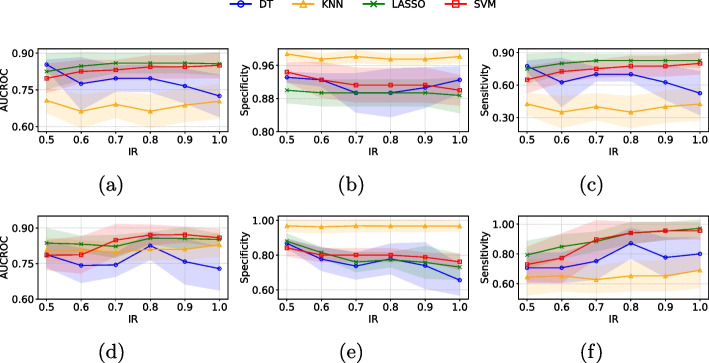
Mean ± standard deviation of the classification metrics (AUCROC, Sensitivity and Specificity) on 5 test subsets using different IR for the PH2 dataset when considering *image features* (first row) and *image embeddings* (second row)

In this section, we present the classification results after selecting relevant features with ensemble FS methods and applying oversampling to the minority class (‘melanoma’ class) using CTGAN for the PH2 and Derm7pt datasets. Figure 5 shows the classification results obtained using *image features* and *image embeddings* extracted from the PH2 dataset. Across both data representations, the optimal IR for enhancing model performance generally ranged from 0.7 to 1.0, depending on the specific metric evaluated. For *image features* (see Fig. 5 (first row)), LASSO with IR=0.8 demonstrated superior performance, achieving the highest AUCROC (0.86) and sensitivity (0.825), while KNN with IR=1.0 exhibited the best specificity (0.923). Regarding *image embeddings* (see Fig. 5 (second row)), SVM with IR=0.9 achieved the highest AUCROC (0.872). KNN with IR=0.7 exhibited the highest specificity (0.969), while LASSO with IR=1.0 attained the highest sensitivity (0.971).

**Fig. 6:**
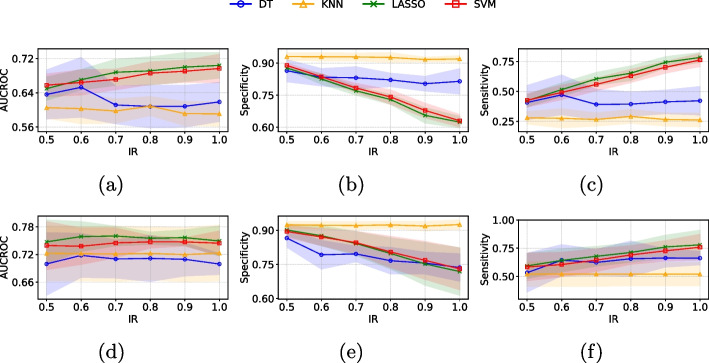
Mean ± standard deviation of the classification metrics (AUCROC, Sensitivity and Specificity) on 5 test subsets using different IR for the Derm7pt dataset when considering *image features* (first row) and *image embeddings* (second row)

Figure 6 shows the classification results obtained using the *image features* and *image embeddings* extracted from the Derm7pt dataset. For *image features* (see Fig. 6 (first row)), LASSO with IR=1.0 demonstrated superior performance, achieving the highest AUCROC (0.704) and sensitivity (0.784). In contrast, KNN with IR=0.5 exhibited the best specificity (0.930). Regarding *image embeddings* (see Fig. 6 (second row)), LASSO with IR=0.7 achieved the highest AUCROC (0.760) and LASSO with IR=1.0 attained the best sensitivity (0.780). KNN with IR=1.0 exhibited the highest specificity (0.925).

Based on the results from both the PH2 and Derm7pt datasets, increasing the IR enhances sensitivity by providing the model with more examples of the minority class, thereby improving its ability to detect these less frequent cases. With a low IR, the model primarily learns to classify the majority class, resulting in high specificity but lower sensitivity. As the IR increases, the model becomes better at distinguishing both classes, which boosts sensitivity but may reduce specificity due to more false positives. Additionally, AUCROC generally increases with IR and stabilizes at higher levels, indicating improved overall class distinction. These findings suggest that a moderate amount of synthetic data can enhance performance metrics without leading to overfitting. Finding the right IR to optimize sensitivity, specificity, and AUCROC is crucial for effective model performance, while also maintaining the model’s generalizability across new data. For a more detailed visualization of additional performance metrics, such as accuracy and F1-score for both the PH2 and Derm7pt datasets, please refer to the Supplementary Material.

### Interpretability methods for identifying melanoma

**Fig. 7:**
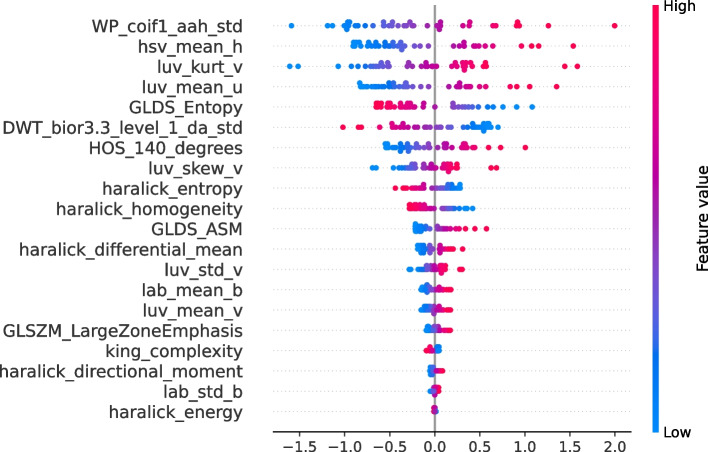
SHAP swarm plot for LASSO model obtained using CTGAN with IR=0.8 and considering the PH2 dataset

To gain interpretability on the trained models and identify the most important geometric, color, and texture features (*image features*) for melanoma classification, we considered SHAP and $CI_\Delta$ for each feature using a hypothesis test based on bootstrap resampling. The features are sorted according to SHAP feature importance, with the horizontal dots representing the SHAP value for a particular feature, and dots on the left and right indicating contributions to a specific class (‘not melanoma’ or ‘melanoma’). The LASSO model with IR=0.8 was selected for the PH2 dataset because it achieved the highest AUROC values for melanoma classification. By observing the most relevant features using this dataset (see Fig. 7), we identified the following texture and color features: WP_coif1_aah_std, hsv_mean_h, luv_kurt_v, luv_mean_u, and GLDS_Entropy. Moreover, high values of all color-related features (hsv_mean_h, luv_kurt_v, luv_mean_u) were associated with the prediction of ‘melanoma’ lesions, whereas low values of these features were linked to ‘not melanoma’ lesions. It is observed that high and low values of WP_coif1_aah_std identify ‘melanoma’ lesions and ‘not melanoma’ lesions, respectively. WP_coif1_aah_std is a feature of WP decomposition, which performs a multiresolution analysis of the image [133]. This could lead to certain features calculated with WP_coif1_aah_std being higher for ‘melanoma’ lesions, especially if ‘melanoma’ lesions exhibit distinctive details at these frequencies. These results are noteworthy because this variable is sensitive for identifying image features with abrupt changes (high-frequency details) [133], which is why higher values are associated with ‘melanoma’ lesions. On the contrary, higher values of GLDS_Entropy are effective for identifying ‘not melanoma’ lesions, while lower values are more efficient at detecting ‘melanoma’ lesions. Entropy can be used to measure variability in the texture of images [134]. A higher entropy indicates greater variability in the texture of an image, whereas a lower entropy indicates a uniform texture [134]. The greater variability in texture in images of ‘not melanoma’ lesions could be explained by the presence of atypical nevi in this class, which could influence the appearance of certain features resembling melanoma-type lesions.

**Fig. 8:**
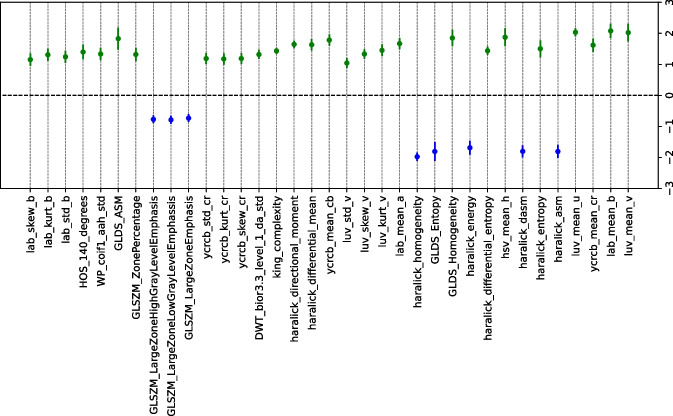
Average differences and the confidence interval ($CI_{\triangle }$) of the bootstrap method by identifying differences between ‘melanoma’ and ‘not melanoma’ lesions for the 35 features *image features* of the PH2 dataset. (Blue: ‘not melanoma’ lesions; Green: ‘melanoma’ lesions)

The statistical significance of the features identified by SHAP (see Fig. 7) is evaluated through a hypothesis test based on bootstrap resampling. Figure 8 shows that the most relevant features provided by SHAP are significant in this hypothesis test. This method identified the statistical relevance of these features in predicting ‘melanoma’ and ‘not melanoma’ lesions. All color features were significant for identifying ’melanoma’, while texture features were mainly relevant for detecting ’not melanoma’ lesions. In previous studies [15, 17], various texture and color features were extracted to detect skin lesions. However, there has been little discussion regarding the impact of the type of texture/color features on melanoma identification. Therefore, it is important to highlight the results obtained, as we have identified several relevant features for predicting ‘melanoma’ and ‘not melanoma’ lesions in the PH2 dataset and determining which features are more relevant for each of the two classes.

**Fig. 9:**
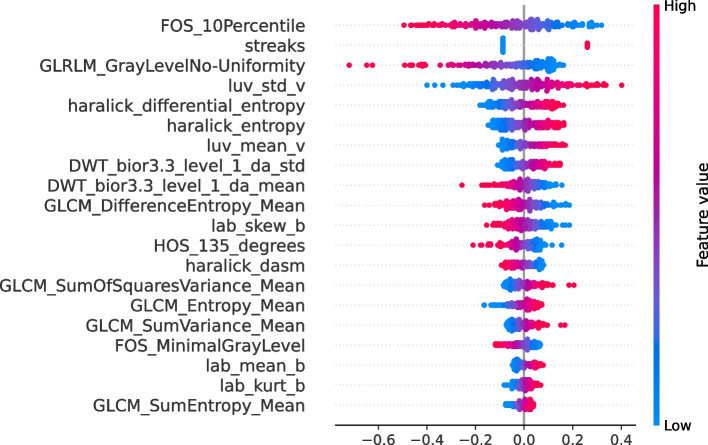
SHAP swarm plot for LASSO model obtained using CTGAN with IR=1.0 and considering the Derm7pt dataset

**Fig. 10:**
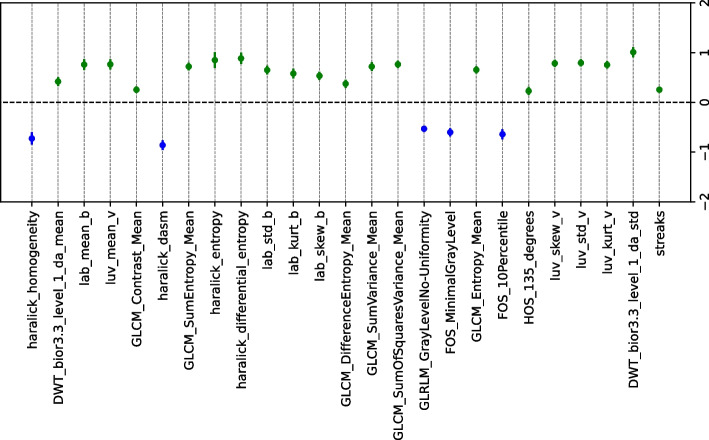
Average differences and confidence interval ($CI_{\triangle }$) of bootstrap method by identifying differences between ‘melanoma’ and ‘not melanoma’ lesions for the Derm7pt dataset. (Blue: ‘not melanoma’ lesions; Green: ‘melanoma’ lesions)

Figure 9 shows the five most relevant features for model predictions using the Derm7pt dataset: Fos_10Percentile, GLRLM_GrayLevelNo_Uniformity, streaks, luv_std_v, and haralick_differential_entropy. LASSO with IR=1.0 was selected based on the best results for melanoma classification in Fig. 6. Fos_10Percentile and GLRLM_GrayLevelNo_Uniformity are features characterized by having higher values for ‘not melanoma’ lesions, whereas lower values characterize ‘melanoma’ lesions. Fos_10Percentile indicates how grayscale pixels are distributed, with a low value in the 10th percentile indicating more uniform textures, whereas higher values identify more varied textures. Similarly to Fos_10Percentile, in GLRLM _GrayLevelNo_Uniformity, high values indicate lower uniformity in the image’s texture. This may appear contradictory, but it is worth noting that images in the ‘not melanoma’ class include benign and malignant lesions, and these may exhibit similar characteristics to those of ‘melanoma’. This explains the presence of irregular textures in dermoscopy images associated with the ‘not melanoma’ class. Similar to the PH2 dataset (see Fig. 7), the color-related features (luv_std_v) were characterized by higher values for ‘melanoma’ lesions than for ‘not melanoma’ lesions. This further reinforces the influence of the presence of different colors on the identification of such lesions. Additionally, high and low values in haralick_differential_entropy impacted the identification of ‘melanoma’ and ‘not melanoma’ lesions, respectively. Haralick features consistently excel in the identification of textural irregularities in ‘melanoma’ lesions as opposed to uniformity in ‘not melanoma’ lesions. In summary, Haralick focused on the local patterns and relationships of grayscale levels, GLRLM captured the length and direction of sequences of grayscale levels, and the FOS percentile evaluated the overall distribution of grayscale levels. The significance of these features was evaluated through the bootstrap resampling test, and all of them satisfied hypothesis $\mathcal {H}_{1}$, i.e., they were statistically significant (see Fig. 10). All features related to the color of the lesion and those that extract irregularities in texture were significant for identifying ‘melanoma’ lesions. The remaining texture-related features found to be relevant with the SHAP method are significant for ‘not melanoma’ lesions.

The analysis using SHAP values and statistical significance testing has provided valuable insights into the roles of geometric, color, and texture features in melanoma classification for both the PH2 and Derm7pt datasets. Our findings reveal that color features are particularly effective in distinguishing ‘melanoma’ lesions, indicating that these characteristics are crucial for identifying malignant cases. Conversely, texture features have shown greater relevance for identifying ‘not melanoma’ lesions. This refined understanding offers clinicians clearer insights into the most significant features for ‘melanoma’ versus ‘not melanoma’ classification. By highlighting the relative importance of color and texture features, our study enhances the interpretability of the model and supports more informed, clinically relevant decision-making. This advancement is essential for improving diagnostic accuracy and reliability in dermatology.

**Fig. 11:**
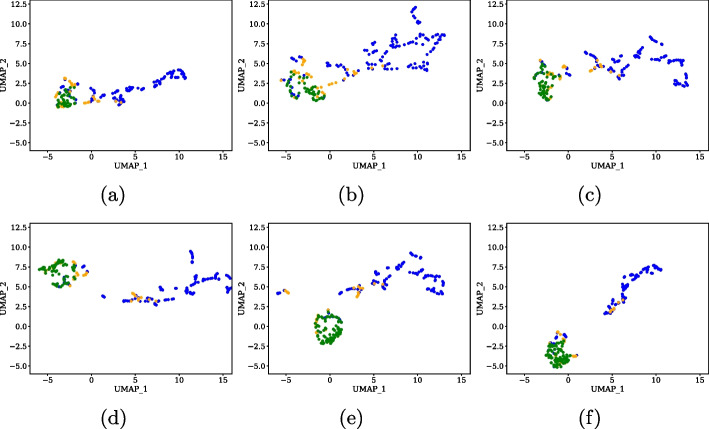
UMAP projections associated with *image features* of the PH2 dataset and using different IR values. **a** IR=0.5; **b** IR=0.6; **c** IR=0.7; **d** IR=0.8; **e** IR=0.9; **f** IR=1.0. (Blue: ‘not melanoma’ lesions; green: synthetic ‘melanoma’ lesions; orange: real ‘melanoma’ lesions)

To visually compare the *image features* associated with ‘melanoma’ and ‘not melanoma’ lesions, UMAP [56] was used. Figure 11 shows projections of *image features* of the PH2 associated with real ‘not melanoma’ lesions (blue), real ‘melanoma’ lesions (orange), and synthetic ‘melanoma’ lesions (green) by varying the IR used. Note that UMAP projections are only displayed for the LASSO model trained with synthetic samples generated by CTGAN for image features from the PH2 dataset, because it is the model that showed the best predictive results. It is observed how the groups of ‘melanoma’ and ‘not melanoma’ lesions separate more distinctly in the UMAP space as you increase the IR in the synthetic ‘melanoma’ samples. This suggests that the increase in the number of synthetic ‘melanoma’ samples helped the model to better distinguish between ‘melanoma’ and ‘not melanoma’ lesions in the reduced feature space created by UMAP. The separation between the groups in the UMAP space indicates improved discrimination between classes, which is essential for effective melanoma detection. However, it is also important to note that as the IR increases, there is an increased overlap between the synthetic ‘melanoma’ samples and the real ‘melanoma’ samples. If the synthetic samples become nearly identical to the real samples, the diversity in the dataset can be lost. This means that the model may lose its ability to learn and generalize effectively. Therefore, it is crucial to strike a balance in the synthetic data generation.

## Discussion

In this study, we evaluate the effectiveness of ensemble FS methods and tabular GAN-based models in improving the identification of melanoma. To characterize the underlying characteristics of the skin lesions, color and geometric features were automatically extracted, as well as different first-, second-, and higher-order texture features. To address the challenges caused by high-dimensionality, ensemble FS was performed by selecting the most relevant features for melanoma identification. Once the best features were chosen, synthetic samples were generated using CTGAN for the minority class (‘melanoma’). We selected CTGAN over other generative models, such as stable diffusion, because it offers superior performance in generating realistic tabular data, which is critical for maintaining the fidelity of minority class representations in imbalanced datasets [36]. Stable diffusion and other generative models primarily excel in generating unstructured data like images or text, but they lack the specialized mechanisms needed to generate tabular data, particularly when it involves mixed-type data and complex relationships between features. These benefits have motivated the selection of CTGAN as oversampling method and to address the CIP. In particular, the amount of synthetic samples created was then determined through different values of the IR. The impact of data augmentation and ensemble FS (with Relief as the *base feature selector*) was evaluated on two dermoscopy datasets: Derm7pt and PH2. CTGAN showed to be effective in generating high-quality synthetic data, and these data generated in conjunction with real data helped to improve the performance of ML-based models in melanoma identification.

One of the main contributions of this study was the introduction of several texture features (including, first, second, and higher-order features) for melanoma identification. Furthermore, we extracted other types of features for melanoma identification, such as color and geometric features. This, along with the interpretability methods, led to the extraction of the most relevant features for melanoma detection in two different datasets composed of dermoscopy images. ‘Melanoma’ and ‘not melanoma’ skin lesions show distinct texture patterns, for instance, dissimilar gray level intensity values. Texture features distinguish one pattern from another and quantify the texture present in the skin lesions. Based on our research, we identified that ‘melanoma’ lesions are mainly characterized by features related to lesion color, whereas ‘not melanoma’ lesions are characterized by texture characteristics. The experimental results demonstrated the improvement in the models through the creation of synthetic samples. Furthermore, this study represents a step forward for early melanoma diagnosis, which, in conjunction with the interpretability capacity of our models, allowed us to determine the most important factors in melanoma detection.

Our findings regarding the CIP in melanoma detection are not only relevant to this specific application but can also be generalized to other scenarios where class imbalance is a significant issue. In many medical and non-medical domains, the scarcity of samples in the minority class poses a similar challenge, affecting the performance of ML-based models [135]. The approach we have developed, which includes the use of data augmentation with GANs and FS techniques, can be adapted to other contexts where the minority class is underrepresented. Specifically, by generating synthetic samples for the minority class, our methodology helps to balance the dataset and enhance model performance. This generalization suggests that our methodology could potentially improve classification performance in various CIP scenarios beyond melanoma detection.

In future work, we plan to extend our methodology by considering multimodal fusion, and combining dermoscopic criteria, patient data (e.g., demographics), image extracted features and image embeddings obtained using different CNN-based models. This extension aims to enhance melanoma identification by leveraging the complementary information from different data modalities. Additionally, we will explore the effectiveness of advanced generative models designed specifically for generating tabular data, and more particularly those based on transformers [136] and diffusion models [43, 45, 137, 138]. Although CTGAN has shown great performance in generating high-quality synthetic data from mixed-type features obtained from dermoscopy images, it is convenient to highlight the potential of recent transformer-based and diffusion-based models for data augmentation, which can be further evaluated in a future work. In a similar way, an ensemble approach by combining multiple classification models may be taken into account to increase the generalization and the predictive results. Another area of research could focus on combining other types of FS methods to deal with high-dimensionality. In this study, ensemble FS methods based on filter techniques were employed for reducing dimensionality and improving predictive results. A new line of research can address the combination of both filter and wrapper methods (discarded in this study because they are time-consuming and highly dependent on predictive models), and solve the computational complexity of wrapper methods using advanced optimization algorithms (e.g., swarm intelligence-based FS methods). Finally, new studies are also necessary to assess the scalability and clinical applicability of our approach across other skin image datasets and to classify other skin cancer types.

## Conclusion

In this study, we evaluated the use of ensemble FS methods along with oversampling techniques to identify melanoma in imbalanced datasets consisting of dermoscopy images (PH2 and Derm7pt datasets). The use of synthetic data created using CTGAN proved to be beneficial for improving melanoma identification, achieving AUCROC values of 87% (with SVM and IR=0.9) and 76% (with LASSO and IR=1.0) for the PH2 and Derm7pt datasets, respectively. Specifically, the supervised linear models SVM and LASSO obtained the highest predictive results. Interpretability methods supported the identification of the most relevant geometric, color, and texture features for melanoma classification. The experimental results demonstrated the capability of synthetic data to help in the development of more generalizable models for melanoma identification.

## Supplementary Information

Supplementary Material 1.

## Data Availability

The datasets used in this study are publicly available.

## Abbreviations

AUCROC: Area under the receiver operating characteristic curve
ANN: Artificial neural network
CIP: Class imbalance problem
CNN: Convolutional neural network
CTGAN: Conditional tabular generative adversarial network
DL: Deep learning
DT: Decision tree
DWT: Discrete wavelet transform
FDTA: Fractal dimension texture analysis
FOS: First-order statistics
FS: Feature selection
GAN: Generative adversarial network
GLCM: Gray-level co-occurrence matrix
GLDS: Gray-level difference statistics
GLRLM: Gray-level run length matrix
GLSZM: Gray-level size zone matrix
HOS: Higher-order spectra
IoU: Intersection over union
IR: Imbalance ratio
KNN: K-nearest neighbour
LASSO: Least absolute shrinkage and selection operator
LBP: Local binary patterns
MAEP: Mean absolute error probability
ML: Machine learning
MRMR: Minimum redundancy maximum relevance
RSVR: Repeated sample vector rate
SFM: Statistical feature matrix
SHAP: Shapley additive explanation
SMOTE: Synthetic minority oversampling technique
SVM: Support vector machine
TVAE: Tabular variational autoencoder
UMAP: Uniform manifold approximation and projection
WP: Wavelet packet
XAI: eXplainable artificial intelligence

## Code availability

Source code is available in the Github repository: github.com/ai4healthurjc/ensemble-fs-aug-melanoma.git.
