# Supplementary material for: Ensemble feature selection and tabular data augmentation with generative adversarial networks to enhance cutaneous melanoma identification and interpretability
Source: BioData Min. 2024 Oct 30;17:46. doi: 10.1186/s13040-024-00397-7 (PMC11526724; doi:10.1186/s13040-024-00397-7)
Supplement: Supplementary file 1 — Supplementary Material 1. [file 13040_2024_397_MOESM1_ESM.pdf]

Supplementary Material: Ensemble feature selection and tabular data augmentation with generative adversarial networks to enhance cutaneous melanoma identification and interpretability

Vanesa Gómez-Martínez[1*], David Chushig-Muzo[1], Marit B. Veierød[2], Conceição Granja[3], Cristina Soguero-Ruiz[1]

[1*]Department of Signal Theory and Communications, Telematics and Computing Systems, Rey Juan Carlos University, Madrid, 28943, Spain.
[2]Oslo Centre for Biostatistics and Epidemiology, Department of Biostatistics, Institute of Basic Medical Sciences, University of Oslo, Norway.
[3]Norwegian Centre for E-health Research, University Hospital of North Norway, Tromsø, 9019 Norway.

*Corresponding author(s). E-mail(s): vanesa.gomez@urjc.es;
Contributing authors: david.chushig@urjc.es; m.b.veierod@medisin.uio.no; conceicao.granja@ehealthresearch.no; cristina.soguero@urjc.es;

1 Melanoma classification by combining ensemble FS methods and data augmentation

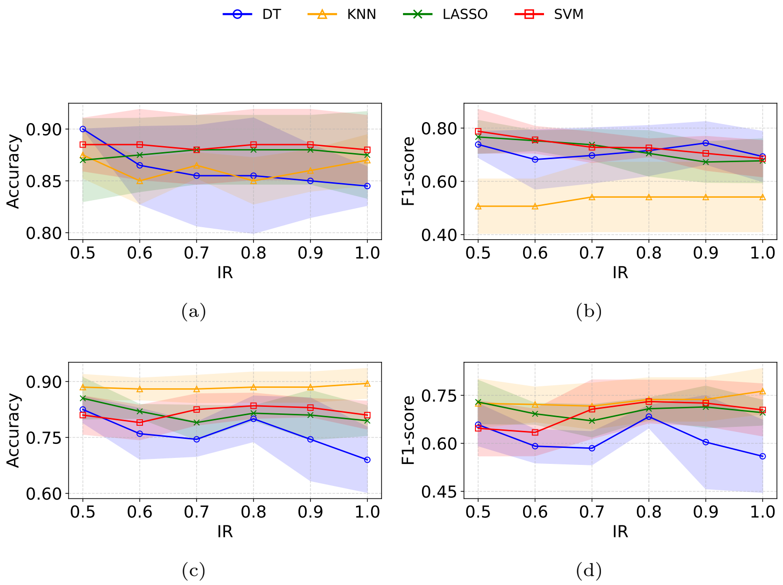

Fig. 1: Mean ± standard deviation of the classification metrics (Accuracy and F1-score) on 5 test subsets using different IR for the PH2 dataset when considering *image features* (first row) and *image embeddings* (second row).

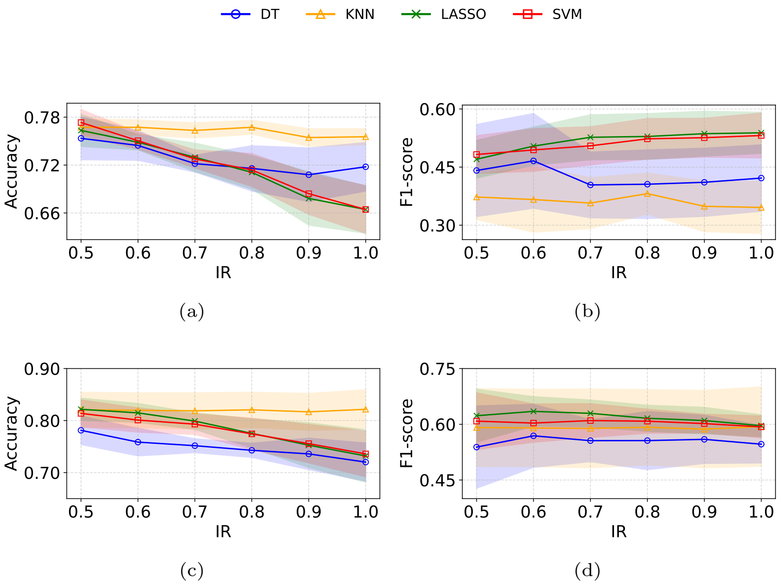

Fig. 2: Mean ± standard deviation of the classification metrics (Accuracy and F1-score) on 5 test subsets using different IR for the Derm7pt dataset when considering *image features* (first row) and *image embeddings* (second row).
